# A Wnt/beta-Catenin Pathway Antagonist Chibby Binds Cenexin at the Distal End of Mother Centrioles and Functions in Primary Cilia Formation

**DOI:** 10.1371/journal.pone.0041077

**Published:** 2012-07-20

**Authors:** Nathan Steere, Vanessa Chae, Michael Burke, Feng-Qian Li, Ken-Ichi Takemaru, Ryoko Kuriyama

**Affiliations:** 1 Department of Genetics, Cell Biology, and Development, University of Minnesota, Minneapolis, Minnesota, United States of America; 2 Graduate Program in Genetics, Stony Brook University, Stony Brook, New York, United States of America; 3 Medical Scientist Training Program, Stony Brook University, Stony Brook, New York, United States of America; 4 Department of Pharmacological Sciences, Stony Brook University, Stony Brook, New York, United States of America; The Hong Kong University of Science and Technology, Hong Kong

## Abstract

The mother centriole of the centrosome is distinguished from immature daughter centrioles by the presence of accessory structures (distal and subdistal appendages), which play an important role in the organization of the primary cilium in quiescent cells. Primary cilia serve as sensory organelles, thus have been implicated in mediating intracellular signal transduction pathways. Here we report that Chibby (Cby), a highly conserved antagonist of the Wnt/β-catenin pathway, is a centriolar component specifically located at the distal end of the mother centriole and essential for assembly of the primary cilium. Cby appeared as a discrete dot in the middle of a ring-like structure revealed by staining with a distal appendage component of Cep164. Cby interacted with one of the appendage components, Cenexin (Cnx), which thereby abrogated the inhibitory effect of Cby on β-catenin-mediated transcriptional activation in a dose-dependent manner. Cby and Cnx did not precisely align, as Cby was detected at a more distal position than Cnx. Cnx emerged earlier than Cby during the cell cycle and was required for recruitment of Cby to the mother centriole. However, Cby was dispensable for Cnx localization to the centriole. During massive centriogenesis in in vitro cultured mouse tracheal epithelial cells, Cby and Cnx were expressed in a similar pattern, which was coincident with the expression of Foxj1. Our results suggest that Cby plays an important role in organization of both primary and motile cilia in collaboration with Cnx.

## Introduction

Centrioles and their morphological equivalent, basal bodies, are complex structures composed of triplet microtubules organized into a 9-fold symmetry. They are essential for cilia/flagella formation and the organization of centrosomes, which serve as primary microtubule-organizing centers (MTOC) in animal cells. To maintain the precise number of MTOCs in cycling cells, the cycle of centriole duplication is tightly coupled with the cell cycle, during which centrioles undergo characteristic morphological transitions, including disengagement (disorientation between two full-length mother and daughter centrioles), nucleation and elongation of procentrioles occurred during G1/S through G2, and separation and maturation at the onset of M phase [1]. When cells become quiescent, mother centrioles convert to basal bodies to organize the primary cilia. Each centriolar event is likely controlled by molecules associated with specific subdomains of the centriole/centrosome.

The distal end of centrioles is associated with a subset of molecules, such as CP110 and Ofd1 [2]–[3]. Since this site corresponds to the growing end of microtubules, these molecules have been shown to be essential for determination of the length of the centriole [3]–[4]. At the end of interphase, a full-length older centriole in the second pair of centrioles has matured by acquiring special structures at its distal end. They are the distal and subdistal appendages [5], and Cep164 [6] and Cenexin (Cnx), an Odf2/Cenexin splicing variant [7]–[8], have been identified as components of the distal and distal/subdistal appendages, respectively. Ninein resides in the subdistal appendage and is responsible for anchoring cytoplasmic microtubules, characteristic functions of the mature mother centriole [9]. The distal end of mother centrioles also serves as the site for initiation of ciliary assembly. Although the majority of distal end-specific components identified thus far have been shown to be essential for cilia formation [3], [6], [10]–[12], it is unknown how individual molecules function during the conversion of centrioles to basal bodies and organization of axonemal microtubules.

Primary cilia serve as a sensory organelle, thus they are linked to the process of signal transduction to mediate communication with the external environment. Much evidence has been accumulated to suggest that cilia play a role in cell signaling in coordination with the sonic hedgehog, planar cell polarity, and platelet-derived growth factor signaling pathways [13]–[14]. In contrast, the involvement of cilia/centrioles in the Wnt/β-catenin signaling seems controversial: while altered cellular responses to the Wnt pathway were noted upon disruption of primary cilia [15]–[17], other reports found that there were no defects in Wnt-dependent developmental processes [18]–[19]. Nonetheless, centriole/centrosome localization of many Wnt signaling molecules, including β-catenin [20]–[21], Dvl [22], Diversin [23], and components of the β-catenin destruction complex [24]–[29], favors the idea of a functional relationship between cilia/centrioles and Wnt signaling.

Here, we identify that Chibby (Cby), a β-catenin-associated Wnt pathway antagonist [30], is essential for cilia formation and specifically localized to the distal tip of the mother centriole distinct from the distal appendage. Cby interacts with Cnx, a component of the appendages, which recruits Cby to the mother centriole and further alleviates the inhibitory effect of Cby on the β-catenin-mediated transcriptional activation in a dose-dependent manner. A close relationship between Cby and Cnx was also shown in in vitro differentiating tracheal epithelial cells during centriogenesis.

## Results

### Cby is Localized at the Distal End of the Mother Centriole

**Figure 1:**
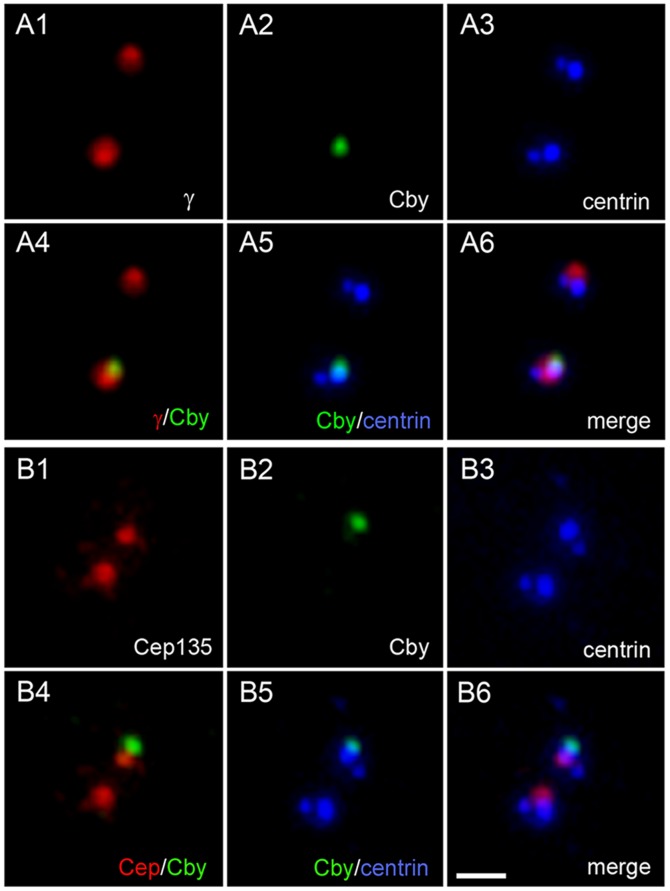
Localization of Cby at one of the centrioles in cultured mammalian cells. HeLa cells expressing GFP-tagged centrin1 were immunostained with Cby and γ-tubulin (A) or Cep135 (B). Cby is detected at one of two centrioles revealed by GFP-centrin. Bar, 1 µm.

**Figure 2:**
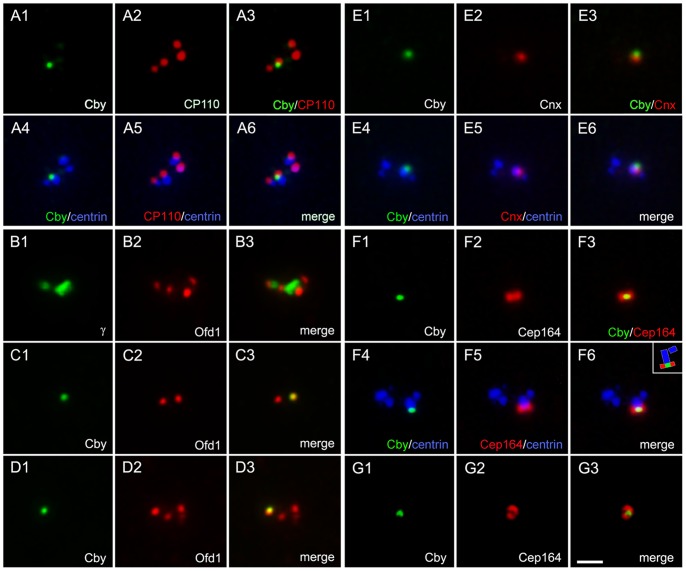
Localization of Cby at the distal end of the mother centriole. RPE1 (A, F–G), CHO (B–D), and HeLa (E) cells were double immunostained with Cby-CP110 (A), Cby-Ofd1 (B–D), Cby-Cnx (E), and Cby-Cep164 (F–G) to show Cby at the distal end of the mother centriole. Both RPE1 and HeLa cells stably expressed GFP-tagged centrin1. The inset (F6) shows a schematic diagram of Cby/Cep164/centrin localization. Bar, 1 µm.

To determine the intracellular distribution of Cby, we immunostained cycling cells with Cby-specific antibodies. All vertebrate cultured cells tested so far from chick to human showed predominantly a single Cby-containing dot. Costaining with centrin and γ-tubulin (Figure 1A) or Cep135 (Figure 1B) shows that these dots corresponded to one of two centrioles of the centrosome. The Cby-containing centriole had a bright centrin signal than the one without Cby and capable of assembling of axonemal microtubules of the primary cilium (see below), indicating that Cby is specifically localized to the mother centriole. Cby did not precisely align with other markers on the centriole. This is particularly prominent with Cep135, which is known to preferentially localize at the proximal end of centrioles [2] (Figure 1B4), suggesting that Cby is present in the distal end. To confirm this, we costained cells with Cby and other markers specific to the distal end of centrioles (Figure 2). CP110 [4] (Figure 2A) and Ofd1 [3] (Figure 2B–D) reside in both mother and daughter centrioles, thus we detected two (Figure 2C2) or four (Figure 2A2, B2, D2) signals in cells at different cell cycle stages. Cby showed better co-localization with these molecules at the end of one of each centrosome. Cnx and Cep164 are appendage components present in the mature centriole. Cby was close but not entirely overlapped with Cnx (Figure 2E). Cep164 is exclusively at the distal appendage, extending outward from each of nine-fold centriolar microtubules. Thus the protein appeared as a ring (Figure 2G2) or a bar (Figure 2F2) at the tip of one of the mother centrioles. Although detected at the same level of the distal position, Cby appeared as a discrete dot in the middle of the Cep164-containing ring/bar (Figure 2F3, G3), suggesting that Cby location is distinct from the distal appendage.

### Cby is Required for Primary Cilia Formation

**Figure 3:**
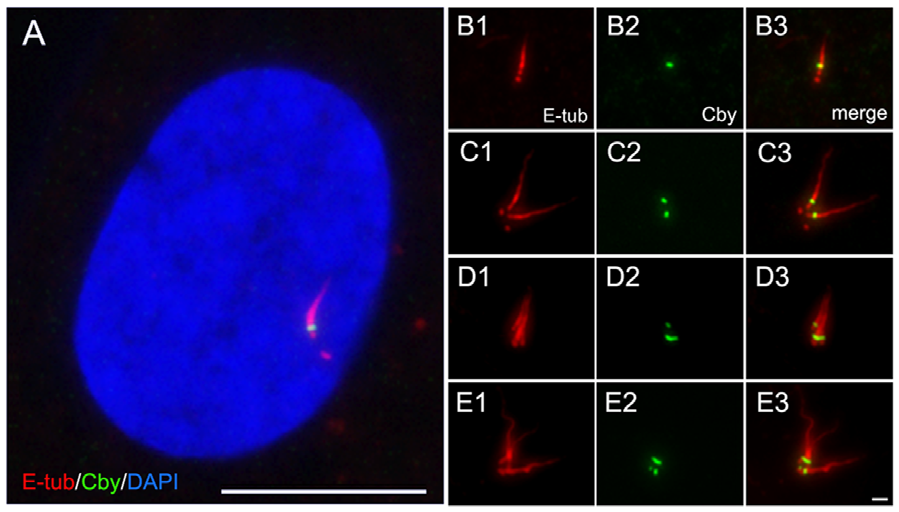
Localization of Cby at the base of primary cilia. Primary cilia induced in quiescent RPE1 cells were stained with Cby and non-tryosinated α-tubulin (E-tub) antibodies. **A** is a merged image with DAPI, and **B–E** show multiple cilia induced by cytochalasin treatment. Bars, 1 µm (B–E) and 10 µm (A).

The majority of distal end-specific centriole proteins identified thus far is known to be involved in the control of primary cilia formation. Cby has also been shown to be essential for the organization of airway motile cilia in mouse models [31]–[32]. To examine further, we immunostained primary cilia induced in human epithelial hTERT-RPE1 cells (RPE1). After 48 hr of serum starvation, almost all cells included a single primary cilium and Cby was detected at the base of each cilium (Figure 3A–B). Figure 3C–E shows multiple primary cilia induced in cells treated with cytochalasin. As a result of cytokinesis inhibition, two pairs of centrioles were left in a single cell where two primary cilia were induced from each mother centriole harboring Cby (Figure 3C). Further inhibition of cytokinesis in successive cell cycles led to the formation of more cilia attached to each Cby-containing site (Figure 3D–E).

**Figure 4:**
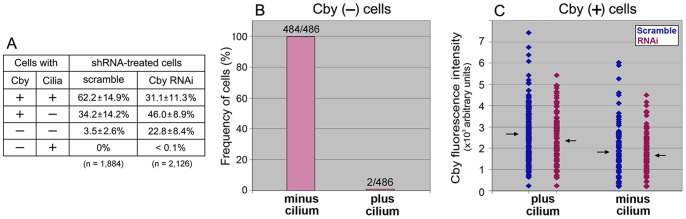
Cilia formation in RPE1 cells expressing different amounts of Cby at the centriole. A: Number of cells with and without Cby and cilia after transfection with scrambled and Cby shRNA. Cells were cultured for 48 hr in a serum-free medium before fixation. Total 1,884 (scramble) and 2,126 (Cby RNAi) cells were examined in repeated six experiments. **B:** Number of Cby-lacking cells with and without cilia after Cby RNAi. No cilia were formed in nearly 100% (484/486) cells expressing non-detectable levels of Cby. **C:** Quantification of the fluorescence intensity of Cby remaining at the centriole in ciliated and non-ciliated cells after transfection with scramble and Cby shRNA. Arrows indicate the position of average fluorescence intensities (266 vs.182 in control and 237 vs. 167 in RNAi cells in arbitrary units with and without cilia, respectively). Cells lacking cilia contained Cby in consistently lower amounts than cells with cilia in both control and RNAi samples.

To determine a role of Cby in primary cilia formation, Cby was depleted from cells by RNAi (Figure 4). Over 20% cells transfected with Cby-specific shRNA lacked Cby (Figure 4A) and almost all of them, if not all (484/486), failed to assemble the cilium (Figure 4B). In control samples transfected with scrambled shRNA vectors, there were also few cells devoid of Cby (∼3%) and cilia were never associated with these cells (Figure 4A). Protein depletion was not always complete and significant numbers of cells still expressed variable amounts of Cby: some were ciliated and others were not (Figure 4A). To correlate the amount of Cby with the presence or absence of cilia, we quantified the fluorescence intensity of Cby in individual cells (Figure 4C). In cells lacking a primary cilium, the level of Cby was consistently lower than in ciliated cells. Control cells treated with scrambled shRNA vectors also showed decreased amounts of Cby at cilia-lacking centrioles. These results suggest that, in order to assemble cilia, cells must accumulate above certain levels of Cby at the distal end of mother centrioles.

### Cby Appears at the Centriole in a Cell-cycle Dependent Manner

**Figure 5:**
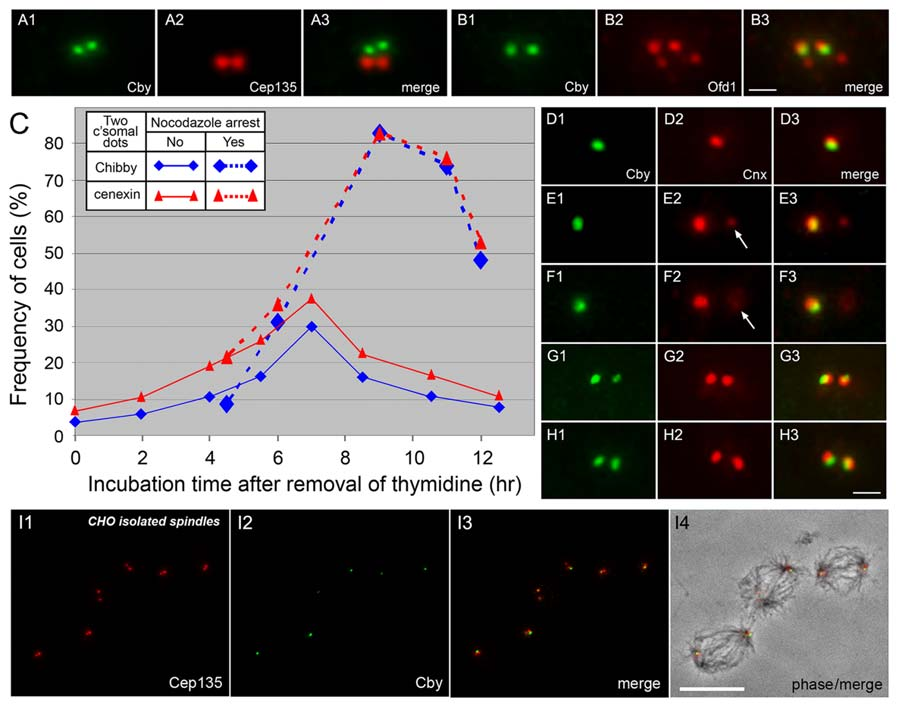
Emergence of Cby at the second site of the centrosome during the cell cycle. A, B: CHO cells double stained with Cby and Cep135 (A) or Ofd1 (B) antibodies show the presence of two Cby-containing centrioles. **C–H:** Time course analysis of Cby/Cnx emergence in partially synchronized CHO cells arrested at G1/S or S phase by thymidine treatment. Two Cby (blue) or Cnx (red) dots were counted after removal of thymidine at time zero (C). Dotted lines indicate cells treated with nocodazole that was added at 4.25 h after washing out thymidine. **D–H** show double staining of centrioles with Cby and Cnx antibodies in cells fixed at 0 (D), 6 (E–F), and 7 (G–H) hr after removal of thymidine. Arrows indicate the position of Cnx-specific centrioles. **I** shows isolated CHO spindles stained with Cep135 and Cby antibodies and a merged image with phase-contrast is shown in **I4**. Cby is seen at one of the two centrioles at each spindle pole. Bars, 1 µm (A–B, D–H) and 10 µm (I).

Although the majority of cells in non-synchronized populations revealed a single Cby-containing centriole, we occasionally noted two Cby signals in one cell (Figure 5A–B). To determine the timing of Cby appearance, we carried out a time course analysis. Figure 5C summarizes the change in the number of cells with two Cby dots (blue diamonds), which was compared with that of Cnx (red triangles), a marker for centriole maturation appearing at the distal end of the mother centriole at the onset of M phase [7]. Over 90–95% of CHO cells that were arrested at G1/S or S by thymidine treatment included a single Cby/Cnx-containing dot (Figure 5D). After washing out the drug (time zero), Cby and Cnx started to appear at the second site of the centrosome with peak levels occurring at ∼7 hr (Figure 5H). Although Cby and Cnx emerged with a similar time course pattern, the number of cells with two Cnx dots always exceeded those with two Cby dots, suggesting that Cnx was recruited to the centriole earlier than Cby. The Cnx-specific signal was weaker and smaller in size than the other associated with both Cby and Cnx (Figure 5E–F). Similarly Cby at the second site was also smaller and less intense than the other (Figure 5G). Thereafter, cells with two dots of Cby and Cnx gradually accumulated during mitotic arrest with nocodazole (dotted lines in Figure 5C). Mitotic cells thus contained a pair of centrioles with associated Cby at each spindle pole (Figure 5I).

### Cnx is Essential for Cby Localization at the Centriole

**Figure 6:**
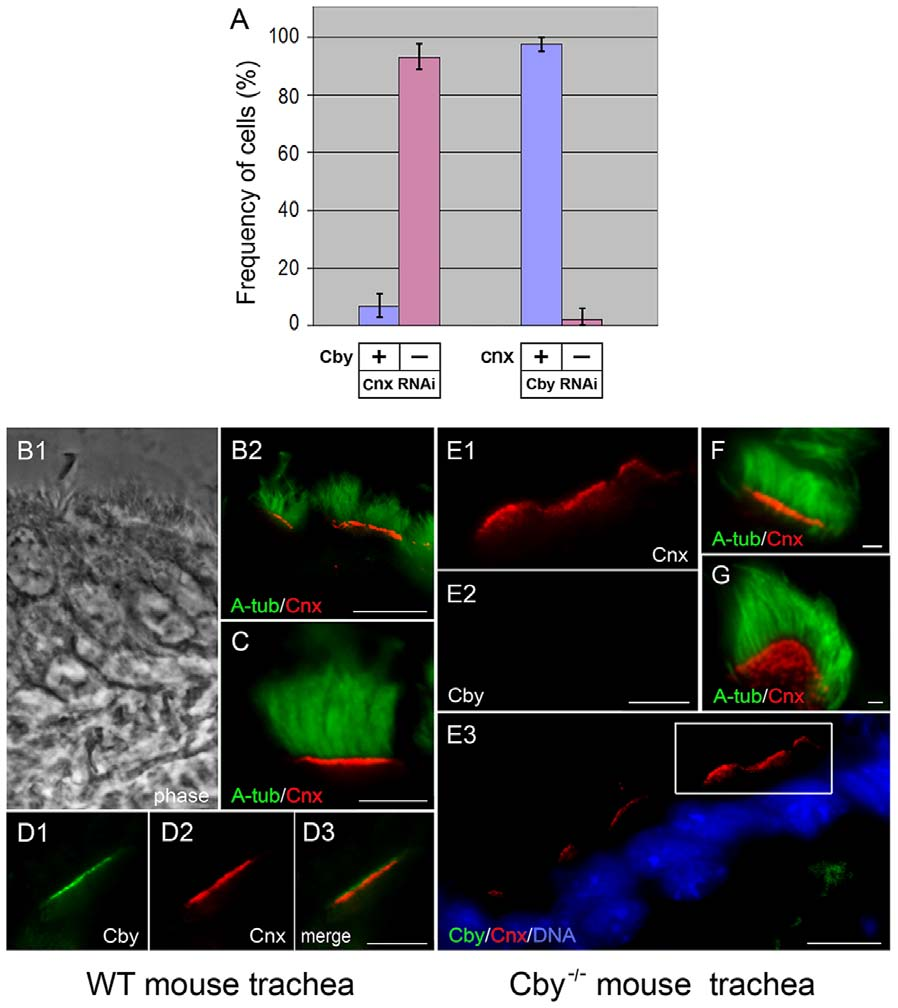
Localization of Cby/Cnx at centrioles/basal bodies in the absence of Cnx/Cby. **A:** Numbers of HeLa cells with (blue) and without (pink) Cby (two columns at left side) and Cnx (two columns at right side) at the centriole after depletion of Cnx and Cby, respectively. **B–G**: Tracheal epithelial tissues of wild type (B–D) and Cby^−/−^ (E–G) mice stained with acetylated α-tubulin (A-tub), Cnx, and Cby antibodies. **B1** is a phase image and **E3** is a merged image with DAPI. A high magnification of the outlined area of **E3** is shown in **E1** and **E2**. In **G**, Cnx-containing basal bodies are seen as individual dots. Bars, 10 µm (B, E3), 5 µm (C–D, E1-E2), and 1 µm (F–G).

The fact that Cnx appeared at the second site of the centrosome earlier than Cby may indicate the importance of Cnx for Cby recruitment to the mother centriole. To test this, we examined Cby in Cnx-depleted cells (Figure 6A): Cby failed to localize to the centrosome in over 95% of the cells lacking Cnx. This was in sharp contrast to Cby RNAi cells where Cnx emerged normally at the centriole (Figure 6A). Thus Cby is likely dispensable for centriole localization of Cnx, which was further confirmed in mouse models. Previous reports showed that Cby was detected at the proximal end of motile cilia in nasal as well as lung airway epithelial tissues [31]–[32]. In cryo-sections of wild-type trachea, we also identified Cnx at the basal bodies, which formed a line to project hundreds of motile cilia into the lumen (Figure 6B–C). Double staining with Cby and Cnx revealed the presence of two distinct lines, and the Cnx-containing line was slightly below the level of the Cby line (Figure 6D). Tracheal sections prepared from Cby^−/−^ mice were shown in Figures 6E–G. Despite the lack of Cby (Figure 6E2), Cnx was detected at the base of these motile cilia as in wild-type trachea (Figure 6E1 and F). The presence of Cnx in individual basal bodies was seen clearly in Figure 6G, where a line of basal bodies became tilted on the plane. From these results, we concluded that Cnx is recruited to centrioles/basal bodies independently of Cby.

### Cnx Interacts with Cby and Alleviates the Inhibitory Effect of Cby on β-catenin-mediated Transcriptional Activation

**Figure 7:**
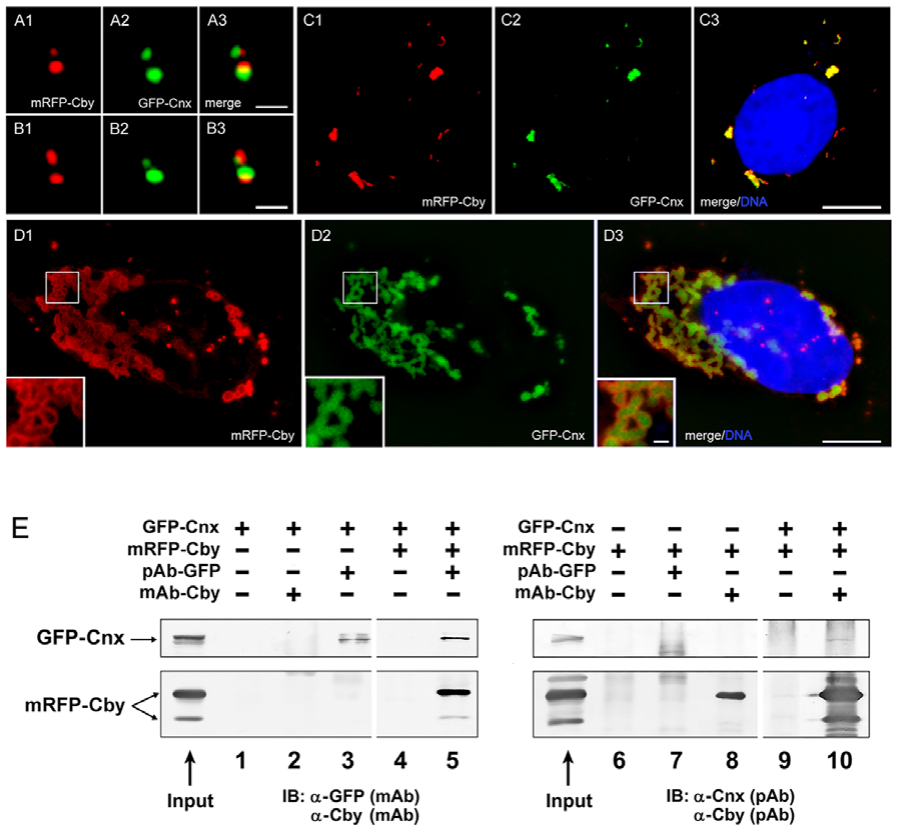
Interaction of Cby with Cnx. **A–D:** U2OS cells expressing mRFP-Cby and GFP-Cnx at the centriole (A–B) and cytoplasmic foci (C–D). Insets of **D** show a high magnification of the outlined area. Bars, 1 µm (A–B, insets of D) and 10 µm (C–D). **E:** Co-immunoprecipitation of mRFP-Cby and GFP-Cnx expressed in HEK293T cells. Proteins were pulled down with polyclonal GFP or monoclonal Cby antibodies and blotted with monoclonal GFP/Cby and polyclonal Cnx/Cby antibodies.

To investigate the relationship between Cby and Cnx, we first expressed tagged exogenous proteins by cotransfection. Figure 7A–B shows cells with mRFP-Cby and GFP-Cnx recruited to the centrosome. Regardless of their expression levels, two proteins showed distinct localization at the centriole. One of the centrioles shown in Figure 7B was labeled with mRFP-Cby more intensely than GFP-Cnx. This centriole may correspond to a mature mother centriole in the older pair of centrioles, which had already been associated with sufficient endogenous Cnx proteins. With increased protein expression, cells started to induce cytoplasmic protein aggregates (Figure 7C–D). In almost all cells, if not all, the aggregates of each protein were exactly co-localized. The formation of cytoplasmic aggregates is common among cells overexpressing centrosomal proteins, such as Cep135 [33]. However, we did not detect exact colocalization of Cby aggregates with other protein aggregates tested thus far, including Cep135, Cep70, SAS6, and Plk4, which may suggest a specific interaction between Cby and Cnx. It is worth mentioning that Cby staining was more intense at the periphery of Cxn aggregates (insets of Figure 7D).

**Figure 8:**
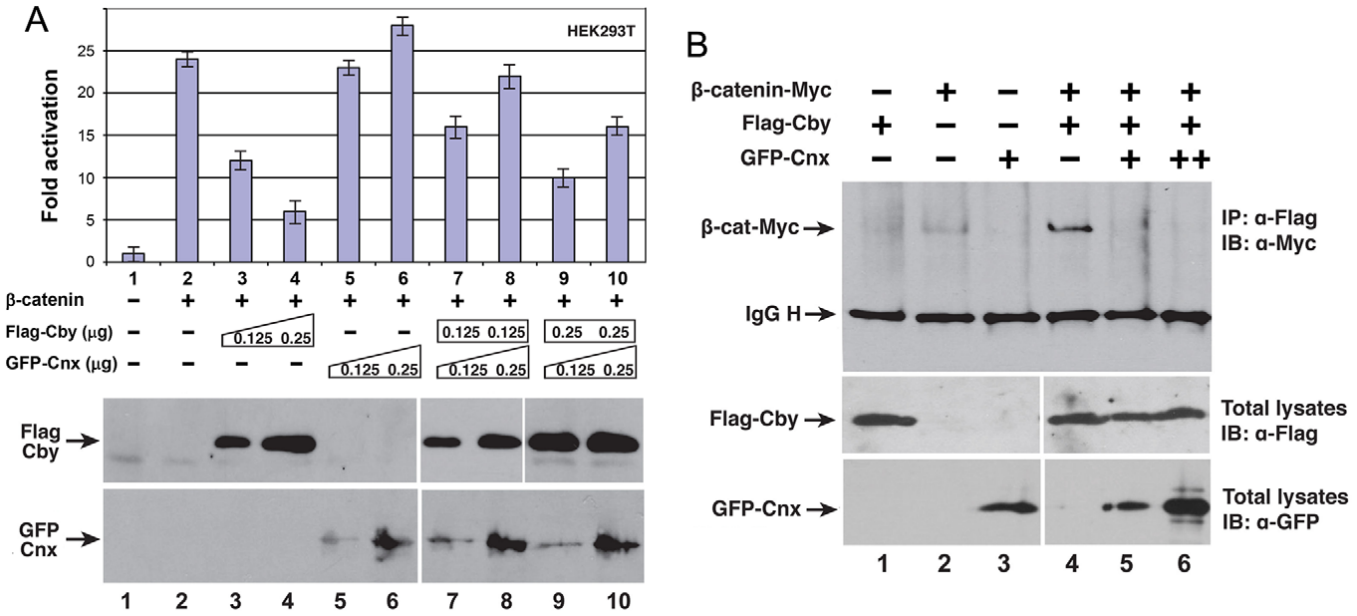
Effect of Cby and Cnx on β-catenin-mediated transcriptional activation. A: HEK293T cells were transfected with a TopFlash luciferase reporter along with stabilized β-catenin, Flag-Cby and GFP-Cnx plasmids. The basal TopFlash value was set as 1. Western blot analysis indicates that Cby and Cnx were stably expressed. **B:** Cnx competes with β-catenin for Cby binding. HEK293T cells were cotransfected with constant amounts of stabilized β-catenin-Myc (1.0 µg) and Flag-Cby (0.5 µg) and increasing amounts of GFP-Cnx (0.5 µg and 1.0 µg). Cell lysates were immunoprecipitated using anti-Flag antibody and detected with anti-Myc antibody. IgG H denotes the IgG heavy chain.

Figure 7E summarizes immunoprecipitation of Cby and Cnx: mRFP-Cby in lysates prepared from cells co-expressing GFP-Cnx was specifically pulled down with GFP antibodies (lane 5). GFP-Cnx remaining in the soluble fraction was also sedimented with Cby antibodies (lane 10). The intensity of immunoprecipitated bands was not always extensive, which may suggest unstable and/or indirect interaction of two proteins. To further confirm molecular interaction between Cby and Cnx, we conducted β-catenin/TCF-dependent luciferase reporter assays [34] (Figure 8). As a β-catenin-associated Wnt pathway antagonist, Cby expressed in HEK293T cells inhibited β-catenin-mediated transcriptional activation (lanes 2 to 4 in Figure 8A). When Cnx was co-expressed, it abrogated the Cby-mediated repression of β-catenin activity in a dose-dependent manner (lanes 7 to 10). Western blot analysis demonstrated that Cby protein was stably expressed in the presence of ectopic GFP-Cnx (Figure 8A). This raises the possibility that Cnx and β-catenin might compete for Cby binding. To test this, we cotransfected constant amounts of Myc-tagged β-catenin and Flag-tagged Cby with increasing amounts of GFP-Cnx, and then immunoprecipitated with anti-Flag antibody and detected β-catenin with anti-Myc antibody. As shown in Figure 8B, coexpression of GFP-Cnx significantly decreased the amount of β-catenin associating with Cby (compare lane 4 to lanes 5 and 6). These results suggest that Cnx and β-catenin form mutually exclusive complexes with Cby, thereby allowing Cnx to alleviate Cby-mediated repression of β-catenin signaling activity.

### Cby and Cnx are Expressed in a Similar Pattern during in vitro Differentiation of Tracheal Ciliated Cells

**Figure 9:**
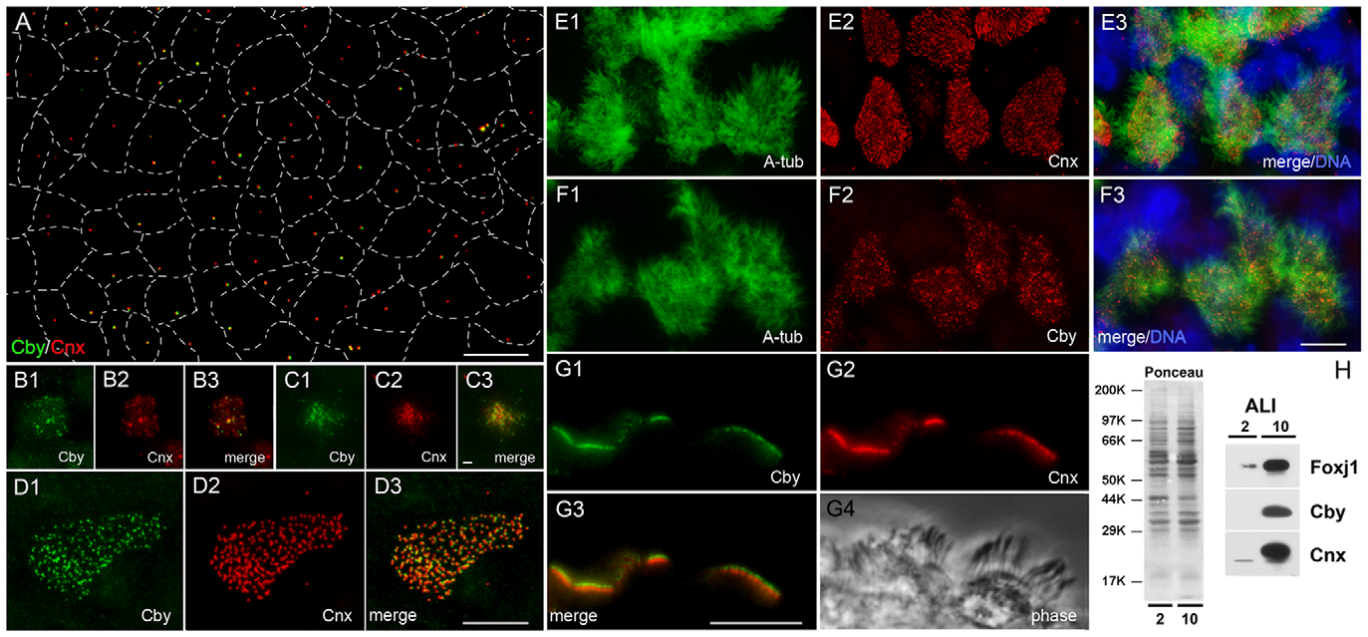
Cby and Cnx in differentiating mouse tracheal epithelial cells (mTECs). **A–G:** mTECs of ALI-0 (A), ALI-2 (B–C), and ALI-10 (D to G) stained with Cby, Cnx, and acetylated α-tubulin antibodies. Merged images with DAPI are shown in **E3** and **F3**, and **G4** is a phase image. Dotted lines in **A** represent borders between adjacent cells. Bars, 1 µm (B–C) and 10 µm (A, D, E–G). **H:** Immunoblot analysis of ALI-2 and ALI-10 mTECs probed with Foxj1, Cby, and Cnx.

To further assess the functional relationship between Cby and Cnx, we analyzed mouse tracheal epithelial cells (mTECs) differentiating in vitro [35] (Figure 9). As previously reported [36], undifferentiated cells covering the entire surface of the membrane contained one or two centrosomes (Figure S1). Although Cnx was found in almost all of the cells, Cby was restricted to only certain numbers of cells at variable fluorescence levels (Figures 9A and S1). After creating an air-liquid interface (ALI), cells initiated massive centriogenesis, leading to the accumulation of Cby and Cnx at the existing centrioles. Simultaneously, a number of small foci appeared in the cytoplasm, in particular around the centrioles (Figure 9B–C). Within 10 to 14 days, fully grown centrioles were formed (Figure 9D), from which hundreds of cilia extended from the apical cell surface (Figure 9E–F). Similar to centrioles/basal bodies in cycling cells (Figures 2E, 5D–H, 7A–B) and in vivo trachea (Figure 6D), Cby was detected more distally than Cnx (Figure 9G). To compare the timing of protein appearance during differentiation, we immunoblotted mTEC lysates prepared at different time points (Figure 9H). Both Cby and Cnx were barely detected in cells at ALI-2 (2 days after creation of ALI), but their amounts significantly increased by ALI-10. These proteins appeared coincidently with the expression of Foxj1, a transcription factor essential for initiation of cilia formation [37], supporting the previous observation that Cby is directly regulated by Foxj1 [32]. From these results, we concluded that Cby and Cnx are expressed in a similar manner during differentiation of ciliated tracheal cells.

## Discussion

This study identifies the Wnt signaling molecule Cby as a centriole protein specifically localized to the distal tip of the mother centriole. The protein showed unique distribution: unlike other distal end-specific components, such as CP110 and Ofd1, Cby is absent in procentrioles and daughter centrioles. The protein is also restricted to the central portion of the ring revealed by a distal appendage component Cep164, which extends outwards from each of the nine doublets of centriolar microtubules [6]. Preliminary studies showed that Cby depletion did not affect Cep164 localization at the mother centriole. These results suggest that Cby is a novel distal end component of the mature mother centriole. The protein may reside in the distal appendage at a restricted area close to the centriolar wall, or it may cap the centriole by sitting on the top of the centriolar microtubules. High-resolution analysis is required to define more detailed localization of Cby at the mature centriole. As the list of the distal end-components specific to the mother centriole is growing [38]–[39], additional molecules showing the same distribution to Cby are likely forthcoming.

We identified Cnx as a possible Cby-interacting molecule. This was further supported by the observation that Cnx alleviated Cby-mediated repression of the β-catenin activity on transcriptional activation in a dose-dependent manner (Figure 8). Cnx is essential for recruitment of Cby to the mother centriole in both cycling and quiescent cells, while Cby is dispensable for Cnx localization at the centriole. Cnx has been reported to be in both the distal and subdistal appendages [10]. Although Cnx and Cby are in close proximity, they do not entirely align, which was also noted in cytoplasmic foci induced by overexpressed exogenous proteins (Figure 7D). As Cnx was consistently detected more proximally than Cby, it is likely that the majority of Cnx may reside in the subdistal appendage. Cnx is known to play a role in the organization and function of the mitotic spindle and spindle poles during cell division [12]. In contrast, Cby does not appear to be involved in the control of centriolar events, other than cilia formation. Many components specific to the distal end of centrioles are important for the control of primary cilia formation. It is thus interesting to know whether these molecules interact, physically and functionally, with each other.

Because of its unique distribution, Cby may function differently from other distal end-specific components during ciliogenesis. In ciliated cells, the distal and subdistal appendages have converted to transition fibers and the basal foot, respectively [40]. Cby is separate from the subdistal structure, implying that the protein may not participate in docking of microtubules to the basal body as proposed for the function of the basal foot [40]–[41]. Transition fibers are believed to anchor the basal body to the membrane at the apical cytoplasm [40]–[42]. Unlike Cep164, Cby appears not to extend outward from the centriolar wall, thus it may not be directly involved in connection of the basal body and membrane. During the early stage of ciliogenesis, the distal end of the mother centriole is capped with a Golgi-derived, concave-convex shaped membrane vesicle [41]. The vesicle becomes indented deeper as the ciliary shaft arises and continues to grow by pushing the vesicle toward the free surface until vesicles fuse with the cell membrane [41]–[42]. There are molecules localized at both the Golgi apparatus and centrosomes. One such protein, Hook2, has recently been shown to play an important role in the formation of membrane vesicles over the top of the mother centriole [43]. Because Cby is in close proximity to the membrane vesicle, it is possible that Cby may be involved in targeting and/or attachment of the vesicles to the centriole in collaboration with vesicle components. Between ciliary axonemes and the centriole is a compartment called the transitional zone, which is essential for the assembly of axonemal microtubules and maintenance of ciliary dynamics [44]. Cby may be a part of the multiprotein complex present at the transition zone and playing an important role in controlling intraflagellar transport and protein sorting of the ciliary membrane [45].

Cby is a β-catenin-binding protein shuttling between the nucleus and cytoplasm [46]. However, with all antibodies we tested to date, we noted detectable levels of Cby only at the centriole in cultured cells. Because nuclear localization of β-catenin is critical for the activation of the Wnt signaling pathway, the amount and intracellular localization of Cby must be tightly controlled by multiple mechanisms [46]–[47]. Therefore, it is likely that nuclear/cytoplasmic Cby is maintained at a low level in cultured cells where the developmental control and homeostatic maintenance of organs and tissues may be unnecessary. Cby physically binds to β-catenin, which is also present in centrosomes [20]–[21]. However, unlike Cby, β-catenin does not appear to preferentially localize at the distal end of the mother centriole. Thus, it is unclear whether Cby and β-catenin are recruited to the centrosome through their interaction with one another. Apparently, many other molecules involved in the Wnt signaling pathway are present in the centriole/centrosome and cilia [22]–[29]. The centrosome may provide a site for Wnt signaling molecules to be sequestered or Wnt/β-catenin pathway components could be involved in the control of centrosomal functions. Further analysis is required to investigate the functional relationship between centrioles/centrosomes and the Wnt/β-catenin pathway.

## Materials and Methods

### Ethics Statement

No approval was required from the University of Minnesota Institutional Review Board for preparation of stable human cell lines derived from de-identified parental hTERT-RPE1 cell lines. All experimental procedures involving mice were approved by the Institutional Animal Care and Use Committee of the Stony Brook University.

### Cell Culture and Transfection

RPE1, U2OS, HEK293T, and GFP-tagged centrin1-expressing HeLa cells (a gift of Dr. A. Khodjakov, Wadsworth Center, Albany, NY) were cultured in DMEM-GlutaMAX (Gibco) supplemented with 10% fetal bovine serum and antibiotics. Stable RPE1 cell lines expressing GFP-centrin1 were prepared by transfection with a plasmid encoding the human centrin1 sequence (Protein ID: ENST00000327228, Open BioSystems). Colonies resistant to G418 at a final concentration of 400 µg/ml were screened by fluorescence microscopy, and then subjected to multiple subcloning cycles.

RPE1 and U2OS cells were seeded on coverslips one day before co-transfection with GFP-Cnx (a gift of Dr. Lee, NIH, MD) and mRFP-Cby using Lipofectamine 2000 (Invitrogen) according to the manufacturer’s protocols. For RNAi, RPE1 and HeLa cells were transfected with a human Cby-specific SureSilencing shRNA or negative control scrambled shRNA plasmid (SABiosciences). Cnx expression was silenced by introducing two types of synthesized target sequences corresponding to nucleotide positions 2138–2156 and 853–871 ligated into pGeneClip vectors (Promega). To confirm Cnx knockdown, RNAi transfection was sometimes repeated 2–3 times.

### Cell Synchronization, Cilia Formation and Spindle Isolation

Cilia were induced in RPE1 cells cultured in a medium lacking fetal bovine serum for 48 hr. For assembly of multiple cilia, cells were first synchronized at G2/M by treatment with RO3306, a Cdk1 inhibitor, for 16 to 24 hr at a final concentration of 10 µM. After washing out the drug, cells were cultured for 1 hr in fresh medium before incubation with 2.5–5 µg/ml cytochalasin D for 2–3 hr. Cells were then treated with a serum-depleted medium to induce primary cilia formation. This process of cytokinesis inhibition and serum starvation was repeated several times to prepare cells with multiple Cby-containing mother centrioles. For synchronization of CHO cells at G1/S or S phase, cells were cultured in Ham’s F-10 medium (Cellgro) containing 5 mM thymidine for 12–16 hr. Mitotic spindles were isolated from synchronized CHO cells at M phase by treatment with thymidine and nocodazole, followed by extraction in a hypotonic medium to release taxol-stabilized spindles [33].

### Immunofluorescence Staining and Quantification

Cells cultured on coverslips were fixed with methanol at −20°C. After rehydration in PBS containing 0.05% Tween-20, cells were immunostained with the following primary antibodies for 30 min at 37°C: mouse monoclonal (Santa Cruz Biotechnology) and rabbit polyclonal Cby [30], rabbit and mouse polyclonal Cep135 [33], monoclonal γ-tubulin (Sigma), polyclonal Cnx (Dr. K. Lee, NIH, MD), polyclonal CP110 (Dr. M. Dias-Bettencourt, IGC, Portugal), polyclonal Cep164 (Drs. E. Nigg, University of Basel, Switzerland and G. Pereira, DKFZ, Germany), polyclonal Ofd1 (Dr. J. Reiter, University of California San Francisco, CA), polyclonal non-tryosinated α-tubulin (Dr. G. Gundersen, Columbia University, NY), monoclonal acetylated α-tubulin (Dr. R. Linck, University of Minnesota, MN) antibodies. Incubation with monoclonal Cby antibodies was carried out at 4°C overnight. After washing off excess antibodies, a mixture of fluorescein-conjugated anti-mouse IgG plus IgM and Texas red-conjugated anti-rabbit IgG antibodies (Jackson ImmunoResearch) was used as secondary antibodies. For triple staining with GFP-centrin, cells were treated with a mixture of Cy3- and Cy5-conjugated mouse and rabbit secondary antibodies.

Microscopic observations were carried out using a Nikon Eclipse microscope with a 100X oil immersion objective (N.A. 1.4) and a Photometrics CoolSNAP camera. 0.1–0.2 µm image slices were merged and further deconvoluted using the SlideBook 4.1 program. To count cells with two Cby and Cnx signals in Figure 5, 100–200 CHO cells were examined at each time point and experiments were repeated at least three times. For quantification of Cby/Cnx/cilia in RNAi cells, we counted 100–150 cells for each sample, repeated in five (Figure 6) and six (Figure 4) experiments. Cby fluorescence was quantified at the centrosome in control and RNAi cells double stained with monoclonal anti-Cby and polyclonal anti-non-tryosinated α-tubulin antibodies. After mounting on a glass slide, the samples were kept in dark for several days to ensure stabilization and equilibration of the mounting medium. Images on same coverslips were captured under identical exposure conditions. Centrosomal spots were designated by creating identical masks and the mean intensity of each spot was measured to compare the Cby intensity in cells with and without the primary cilium.

### Immunoprecipitation and Western Blot Analysis

HEK293T cells co-transfected with GFP-Cnx and mRFP-Cby were washed three times with PBS and collected into ice-cold lysis buffer (20 mM Tris-HCl, pH 7.2, 135 mM NaCl, 1.5 mM MgCl_2_, 1 mM EGTA, 1% Triton X-100, 10% glycerol) supplemented with a cocktail of protease inhibitors. After centrifugation at 12,000 rpm for 10 min at 4°C, the supernatants were co-immunoprecipitated using polyclonal anti-GFP (Invitrogen) or monoclonal anti-Cby antibodies as described previously [47]. After separation on 10% SDS-PAGE, Cby and Cnx bands were visualized by blotting with monoclonal GFP (Roche) or polyclonal Cnx, and monoclonal Cby or polyclonal Cby antibodies, and alkaline phosphatase-conjugated anti-mouse or anti-rabbit secondary antibodies (Hyclone) using BCIP/NBT chromogen [33]. For Immunoprecipitation of β-catenin, Myc-tagged β-catenin was expressed along with Flag-Cby and GFP-Cnx [47], and then pulled down using monoclonal anti-Flag antibody (Sigma). The band was visualized by probing with monoclonal anti-Myc (Invitrogen) and HRP-conjugated secondary antibodies (Jackson ImmunoResearch) [47].

### Preparation of mTEC Cultures and Mouse Tracheal Sections

In vitro cultures of mouse tracheal epithelial cells were prepared from 2–4 month old C57BL/6J wild-type and Cby^−/−^ mice according to the procedure of You et al. [35]. Cells on membranes were fixed in either 3.2% paraformaldehyde with 3.0% sucrose in PBS (pH 7.4) or methanol:acetone (1∶1) on ice for 10 min, and then blocked with PBS-Tween 20 containing 5% BSA and 3% goat serum for 1–2 hr before processing for immunofluorescence staining. For immunoblot analysis, cells on membranes were lysed in a 1∶1 mixture of RIPA and 2X SDS loading buffer, boiled at 95°C for 5 min and stored at –20°C before use. The anti-Foxj1 antibody was a gift from Dr. S. Brody (Washington University, St. Louis, MO). Cryo-sections of tracheal epithelial tissues were prepared from wild-type and Cby^−/−^ mice as described previously [31]–[32]. After post-fixation with cold methanol, sections were immunostained with Cby, Cnx, and acetylated α-tubulin antibodies as above.

### Reporter Assays

TopFlash assays were performed as described previously [47]. Briefly, HEK293T cells in 24-well plates were transfected with 20 ng of TopFlash luciferase reporter with or without 10 ng of an expression vector for stabilized β-catenin (pt β-catenin), and the indicated amounts of plasmids encoding Flag-Cby and GFP-Cnx. Luciferase activity was measured 24 hr post-transfection, and normalized to Renilla luciferase activity used as an internal control. Each transfection was carried out in duplicate and the means ± SD from three separate transfections were calculated.

## Supporting Information

Figure S1 **Immunostaining of undifferentiated mTECs with Cby and Cnx antibodies.** Cells were double stained with Cby/Cep135 (A) and γ-tubulin/Cnx (B) antibodies before exposure to air (ALI-0). Merged images are shown in **A3** and **B3**, and dotted lines indicate cell borders. Almost all cells, if not all, expressed Cnx, but Cby was detected in only a limited number of cells at different levels of fluorescence intensity. Bar, 10 µm.
(TIF)
