# Supplementary figures and images for: A Wnt/beta-Catenin Pathway Antagonist Chibby Binds Cenexin at the Distal End of Mother Centrioles and Functions in Primary Cilia Formation
Source: PLoS One. 2012 Jul 20;7(7):e41077. doi: 10.1371/journal.pone.0041077 (PMC3401179; doi:10.1371/journal.pone.0041077)

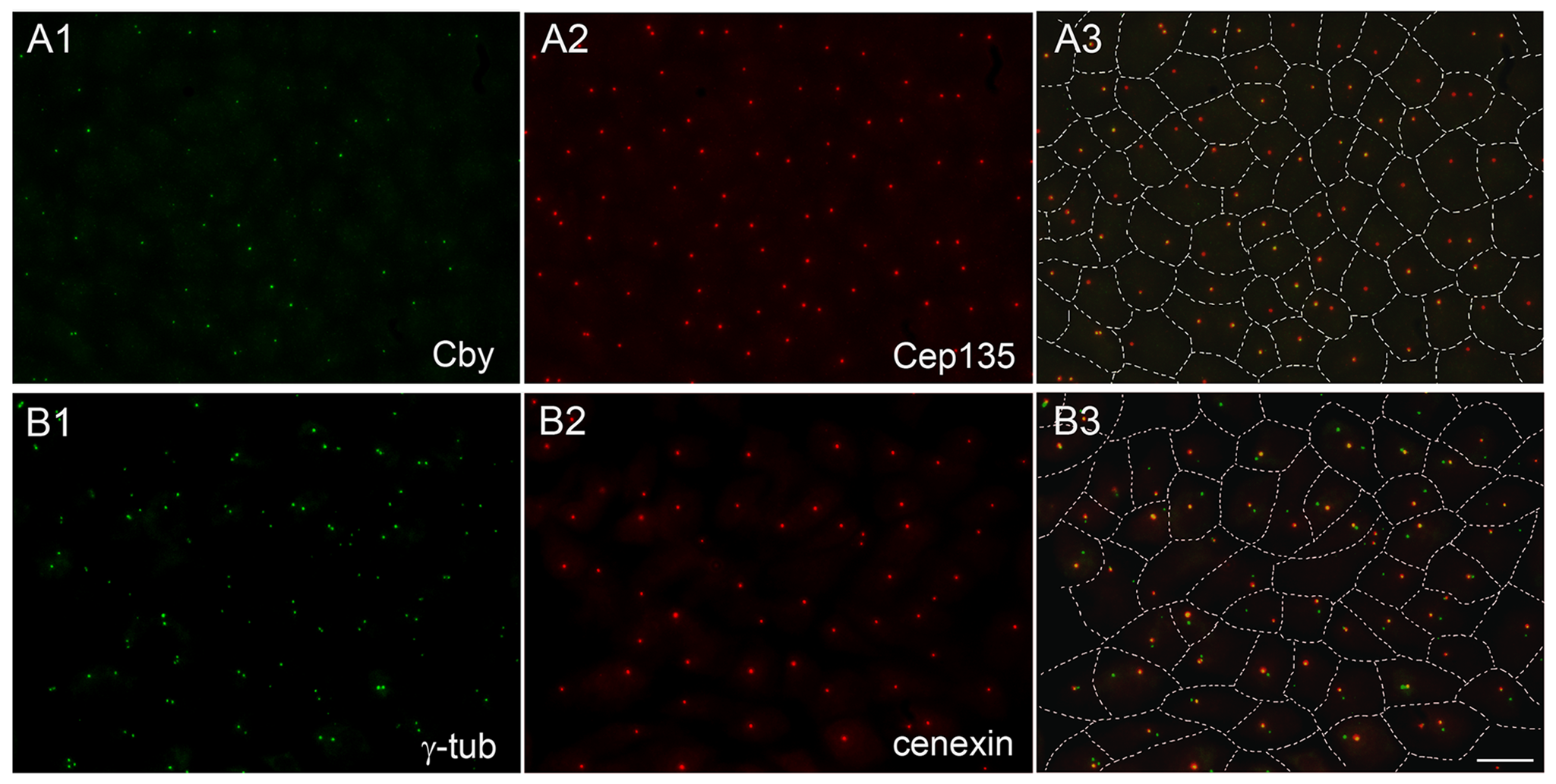

Supplement: Figure S1 — Immunostaining of undifferentiated mTECs with Cby and Cnx antibodies. Cells were double stained with Cby/Cep135 (A) and γ-tubulin/Cnx (B) antibodies before exposure to air (ALI-0). Merged images are shown in A3 and B3, and dotted lines indicate cell borders. Almost all cells, if not all, expressed Cnx, but Cby was detected in only a limited number of cells at different levels of fluorescence intensity. Bar, 10 µm. (TIF) [file pone.0041077.s001.tif]
